# Some Strains of Organisms Found in Dysentery Suspect Cases in France, Showing Peculiar Agglutinating Phenomena and Sugar Reactions

**Published:** 1918-05

**Authors:** 


**Some Strains of Organisms Found in Dysentery Suspect Cases in France, Showing Peculiar Agglutinating Phenomena and Sugar Reactions.** By Captain F. N. B. SMARTT, R. A. M. C., *Journal R. A. M. C.*, February, 1918.

Captain Smartt describes all the organisms which he succeeded in isolating from a number of dysentery suspect cases and which gave agglutination with specific dysentery sera. The organisms were recovered from 18 cases sent down suffering from diarrhea with blood and mucus or with a recent history of such diarrhea. At the time of examination the majority of the stools contained neither blood nor mucus macroscopically and microscopically and no amoebae. The sugar reactions were differentiated into seven types; of these one claimed seven of the total cases and fell into the Flexner-Y group as far as could be ascertained with the available carbohydrates; the remaining six types showed sugar reactions quite dissimilar to any known dysentery group.

The cases were divided into two series. The first series occurred in routine examination over a period of one week, during which time 143 stools were examined. Out of these 31 were returned as positive on the assumed specificity of the agglutination phenomenon. Only three of the seven non-lactose fermenting strains isolated were typical B. dysenteriae. In the second series 27 cases out of 120 were returned as positive on agglutination, but only six strains of the eleven isolated were non-lactose fermenting and of these only four were typical B. dysenteriae.

The author found the fermentation reaction of the sugars with these organisms extraordinarily varied. Even the lactose fermenters did not conform to any special type of intestinal organism. It seemed unreasonable that in a comparatively small number of cases so many different strains, all possessing certain similar specific characters, should be present. Two possibilities suggested themselves : either the atypical strains are specific organisms which have acquired atypical fermentation characters, or they are non-specific organisms of the intestinal tract wich have acquired certain specific properties and fermentation characters atypical of any well-defined non-specific group. All the atypical ncn-lactose fermenting strains conformed in other cultural respects to B. dysenteriae : they did not liquefy gelatine, they were non-motile gram-negative bacilli and gave the characteristic milk reactions of the group — early acid followed by alkalinity. Two of the strains gave the fermentations of the Gaertner group but did not agglutinate with specific Gaertner serum. Sugar fermentation tests were repeated with most of the strains — in some instances six weeks after first isolation — but there was no reversion.

